# Perioperative management of subcutaneous foslevodopa/foscarbidopa: a case report

**DOI:** 10.1007/s00415-025-13149-2

**Published:** 2025-05-21

**Authors:** Samir Abu-Rumeileh, Markus Otto, Michael Bucher, Karl-Stefan Delank, Carmelo Carrubba, Georg Ebersbach, Jan Kassubek, Bettina-Maria Taute

**Affiliations:** 1 Department of Neurology, Martin-Luther-University Halle-Wittenberg, 06120 Halle (Saale), Germany; 2 Department of Anesthesiology and Operative Intensive Care, Martin-Luther-University Halle-Wittenberg, 06120 Halle (Saale), Germany; 3 Department of Orthopedic and Trauma Surgery, Martin-Luther-University Halle-Wittenberg, 06120 Halle (Saale), Germany; 4 Movement Disorders Hospital, Kliniken Beelitz GmbH, 14547 Beelitz-Heilstätten, Germany; 5 Department of Neurology, Ulm University Hospital, 89081 Ulm, Germany; 6 Department of Cardiology, Angiology and Intensive Care Medicine, Martin-Luther-University Halle-Wittenberg, 06120 Halle (Saale), Germany

Dear Sirs,

The management of patients with Parkinson’s disease (PD) undergoing surgery is challenging. Indeed, PD patients have a higher incidence of postoperative complications, longer hospital stays, higher hospital costs, and overall increased mortality [1]. Appropriate pharmacological management with strict adherence to the patient’s individualised medication regimen is essential to preserve patient function and prevent acute complications such as akinetic crisis and dopamine agonist withdrawal syndrome in the perioperative period [2].

Traditional PD medication options in the perioperative setting are several but present some limitations: (1) enteral levodopa via nasogastric tube, which may be affected by reduced absorption in the context of PD gastrointestinal hypomotility (possibly exacerbated by opioid medications) and may be challenging if patients cannot receive oral medication due to fasting or surgical considerations; (2) levodopa–carbidopa intestinal gel, which provides continuous release but requires surgery for percutaneous endoscopic gastro-jejunal tube insertion; (3) transdermal rotigotine and (4) subcutaneous apomorphine infusion, which, especially the latter, may be associated with neuropsychiatric and vegetative adverse events such as nausea and hypotension [2–5].

Foslevodopa/foscarbidopa is a recently introduced hydrophilic levodopa derivate that is administered as continuous subcutaneous dopamine replacement therapy 24 hours a day to patients with advanced PD and motor fluctuations. The hydrophilic nature of the drug leads to a continuous infusion via a pump, resulting in more stable plasma levels of levodopa and, thus, improving motor fluctuations [6–8]. In a phase 3 trial, foslevodopa/foscarbidopa significantly influenced positively motor fluctuations with a decrease in off time (− 3.5 hours) and an increase in on time without troublesome dyskinesia (3.8 hours) [6].

To date, there are no data on the perioperative use of foslevodopa/foscarbidopa. Given the aforementioned limitations of traditional PD medication options, foslevodopa/foscarbidopa may offer some advantages in terms of continuous administration through an accessible and simple route (i.e. subcutaneous). This case report describes the management and outcome of foslevodopa/foscarbidopa administration in a patient undergoing an elective mini-invasive total joint replacement.

The patient was diagnosed with PD at the age of 58 years. Her first motor symptom was rest tremor of the left upper limb, which occurred at the age of 57. Non-motor symptoms included hyposmia, asthenia and insomnia due to nycturia. Medical history revealed arterial hypertension, right coxarthrosis and chronic renal insufficiency due to right nephrectomy for hydronephrosis and left nephrotomy for a renal pelvis cystitis. The symptoms responded well to antiparkinsonian medication (levodopa + benserazide and safinamide) for several years. Wearing off and unexpected OFF episodes began at the age of 60 years, and dyskinesia at age 65 years. The adjustment of anti-PD medication doses and the addition of other drugs (i.e. opicapone, piribedil, and levodopa inhalation powder) were progressively less effective in counteracting the significant motor fluctuations.

Therefore, at the age of 66 years, the patient was admitted to a PD reference centre for the introduction of foslevodopa/foscarbidopa. DBS and continuous infusion levodopa/carbidopa gel were not considered due to the patient’s refusal of invasive device assisted therapies. At the time of admission, Movement Disorders Society-Unified PD Rating Scale (MDS-UPDRS) part III score was 38 (during the off state) and the Hoehn & Yahr stage was 4. The mini-mental state examination showed normal values. The patient was on levodopa 950 mg/day, safinamide 100 mg/day and opicapone 50 mg/day.

Foslevodopa/foscarbidopa was started and progressively adjusted. During the hospitalisation, motor fluctuations improved with disappearance of wearing off and dyskinesia. Unexpected OFF episodes were reduced with bolus administration. No adverse events were reported. The patient was discharged with the following infusion rates: basal infusion rate 0.35 mL/h (59.5 mg/h) during the day (from 6 am to 9 pm), infusion rate during the night 0.22 mL/h (37.4 mg/h), and high rate 0.36 mL/h (61.2 mg/h). At discharge, safinamide 100 mg/day remained the only concomitant medication.

After a few months, the patient was admitted to our hospital for an elective right total hip replacement. The infusion rates on admission were 0.35 mL/h (59.5 mg/h) basal infusion rate, 0.22 mL/h (37.4 mg/h) low/night rate, and 0.36 mL/h (61.2 mg/h) high rate. Considering the above-mentioned limitations of traditional therapeutic options, we decided to continue off-label foslevodopa/foscarbidopa therapy in the perioperative period. Safinamide was not given on the day of surgery. The right upper arm was chosen as the perioperative infusion site for foslevodopa/foscarbidopa. This allowed the pump to be positioned safely outside the operating area. Neurological assessment was performed 1.5 hours before induction of anaesthesia, MDS-UPDRS part III score was 21 (under basal infusion rate). At this time point, the infusion rate was adjusted to the low rate (0.22 mL/h) to account for reduced motor activity under general anaesthesia. The surgical operation was performed under general anaesthesia without complications and took approximately 1 hour. At a later neurological assessment, 2 hours after extubation, the patient showed a slight worsening of motor symptoms, particularly of upper limb tremor, compared with the previous assessment (UPDRS was not calculated due to the patient’s immobility). No delirium, hallucinations or vegetative symptoms occurred. The basal rate was introduced approx. 4–5 hours after extubation. From this time, foslevodopa/foscarbidopa was administered according to the previous schedule. The patient was monitored for 72 hours postoperatively, the clinical picture, in particular the motor symptoms, returned to baseline (preoperative phase) and the patient did not develop delirium and hallucinations throughout the postoperative period. She was discharged with the previous infusion rates and underwent rehabilitation therapy.

By providing continuous dopaminergic stimulation, foslevodopa/foscarbidopa showed significant benefits in terms of motor function and motor complications in our case, as shown in previous clinical trials [6, 7]. First, we achieved a strict adherence to the patient’s individual medication regime by maintaining the basal infusion rate in the preoperative and postoperative phases, with only a shift to the low rate during general anaesthesia and the first perioperative hours. This approach prevented the occurrence of akinetic crisis and other motor complications and maintained a good patient function throughout the perioperative period, as documented by serial neurological assessments. Of note, our patient was still on foslevodopa/foscarbidopa before surgery and did not need a change in treatment option, so, theoretically, given our promising data, it may be interesting to evaluate the perioperative use of foslevodopa/foscarbidopa even in patients who have not previously used it. In this regard, there is a report of a “naïve” patient with an akinetic crisis who was treated off-label with foslevodopa/foscarbidopa and had a good outcome [5]. However, there are still challenges in prescribing and organising foslevodopa/foscarbidopa infusion in a short time. Therefore, this approach would not be feasible in contexts such as emergency surgery, but may be easier to implement in surgical patients already treated with foslevodopa/foscarbidopa and/or in the case of elective surgery.

On another issue, our patient did not experience hallucinations or delirium in the postoperative period despite continuous administration of foslevodopa/foscarbidopa. This is noteworthy given that postoperative delirium in PD has a prevalence of 11% to 60% and is associated with motor severity [2], whereas psychosis or hallucinations were reported in 15%−17% cases of patients on foslevodopa/foscarbidopa in previous clinical trials [6, 7]. Therefore, more data are needed to address the incidence of neuropsychiatric symptoms in this population in the perioperative period.

Another advantage of foslevodopa/foscarbidopa, over other subcutaneous or transdermal anti-PD drugs, such as apomorphine and rotigotine, is the lack of association with hypotension and nausea as adverse events [6, 7].

Given the high risk of postoperative functional deterioration, patients with PD require multidisciplinary management by neurologists, anaesthetists and surgeons. In our case, we reached an interdisciplinary consensus on the best treatment option through team meetings in the perioperative period.

Our case report highlights for the first time (1) the potential role of foslevodopa/foscarbidopa as a valid therapeutic option in the perioperative management of PD patients, especially in previously treated cases and/or in elective surgery, and (2) a favourable side-effect profile of therapy in the perioperative period. However, its applicability in other surgical contexts should be further investigated.
